# Case Report: Unusual persistent elevation of troponin I-systemic sclerosis masked by acute myocardial infarction

**DOI:** 10.3389/fimmu.2026.1675907

**Published:** 2026-02-05

**Authors:** Lixia Zhang, Shuqi Li, Jinglei Niu, Ming Bai

**Affiliations:** 1 Heart Center, The First Hospital of Lanzhou University, Lanzhou, China; 2 The First Clinical Medical School of Lanzhou University, Lanzhou, China

**Keywords:** cardiac troponin I, multiple organ involvement, myocardial infarction, percutaneous coronary intervention, systemic sclerosis heart involvement, systemic sclerosis

## Abstract

Myocardial infarction (MI) is a serious cardiovascular emergency that causes myocardial necrosis due to acute occlusion or spasm of a coronary artery leading to persistent ischemia and hypoxia; it is the major cause of death in patients with cardiovascular diseases worldwide. Systemic sclerosis (SSc) is a rare immune-mediated rheumatic disease characterized by fibrosis of skin and internal organs and vascular lesions. Although the survival in SSc has been improved in the past decades, the mortality rate remains high. Here we report a case of myocardial infarction combined with SSc and document the diagnostic and treatment process. A 55-year-old male patient underwent percutaneous coronary intervention (PCI) because of acute inferior MI, but a downtrend of cardiac troponin I (cTnI) was absent after operation. At 1 month later, the patient was readmitted for treatment due to residual vascular lesions, and the serum cTnI remained persistently high. After a thorough examination and histopathologic biopsy, the patient was ultimately diagnosed with a combination of SSc. Electrocardiograph, medical images, cardiac enzymes, and histopathological changes as well as coronary angiographic findings revealed the severity and complexity of the patient’s condition. At 3 months after the second discharge, the follow-up data showed that the patient’s condition improved significantly. This case may provide a novel perspective on the diagnosis and clinical management of this rare comorbidity.

## Introduction

Cardiovascular disease is the primary cause of death throughout the world; of these, myocardial infarction (MI) accounts for the majority of deaths (1). Acute myocardial infarction (AMI) is a severe event of myocardial necrosis mainly caused by coronary atherosclerosis. Moreover, measurement of cardiac troponin (cTn) is the preferred biomarker method for risk stratification of acute coronary syndromes (ACS) (2). However, in patients undergoing selective percutaneous coronary intervention (PCI) with normal baseline cardiac troponin, post-PCI elevation of troponin is often regarded as associated with procedural complications or new myocardial ischemia (3). Systemic sclerosis (SSc) is a rare immune-mediated conjunctive tissue disease with a complex pathogenesis featured with chronic and progressive skin and organ fibrosis (4). Cardiac involvement is a major disease manifestation of SSc which accounts for a significant portion of mortality related to SSc (5). Nevertheless, early diagnosis of SSc is not easy due to the absence of typical clinical manifestations. Here we reported a middle-aged male patient who underwent emergency PCI for AMI. However, there was no downtrend of serum cTnI after revascularization. Ultimately, the patient was diagnosed with systemic sclerosis and SSc heart involvement during rehospitalization. This rare case may provide clinicians with fresh insights when faced with a similar situation.

## Case presentation

**Figure 1:**
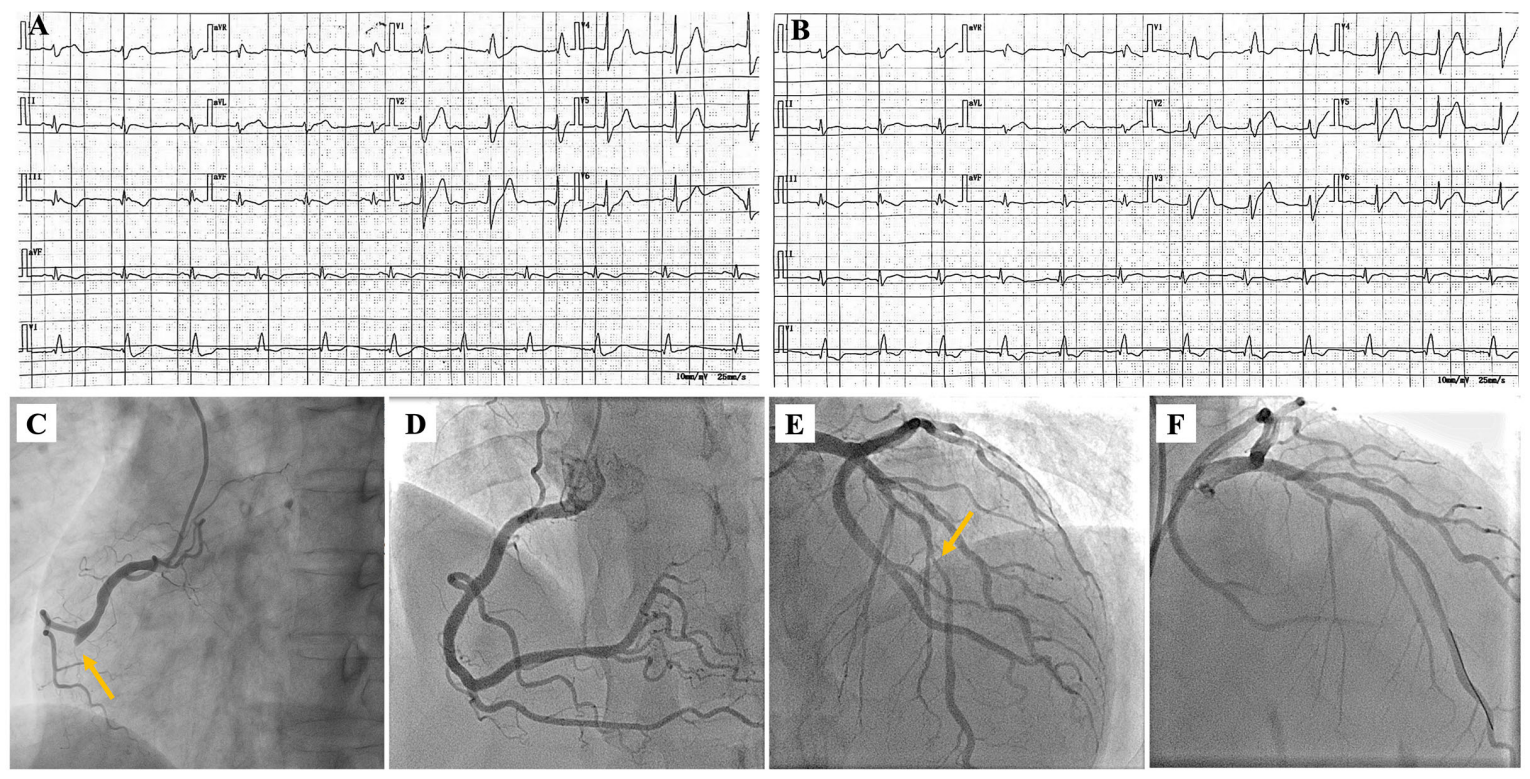
ECG and coronary artery imaging characteristics of the patient at first admission and re-admission. Result of ECG at first admission **(A)**. ECG for re-admission **(B)**. Coronary angiogram (CAG) in left anterior oblique view showing the proximal segmental occlusion at RCA **(C)**. Imaging of stent implantation in RCA **(D)**. CAG in cranial view showing severe stenosis in the middle part of the LAD **(E)**. Image after implantation of one stent in LAD **(F)**.

A 55-year-old male presented to the emergency department with persistent chest pain, breath shortness, and palpitations for 13 h. The patient complained of no specific past medical history except hypertension for 5 years. The consultation revealed that the patient had four cardiovascular risk factors: hypertension, smoking, male, and age >45 years. The electrocardiogram (ECG) showed Q-wave formation, T-wave inversion in II, III, and AVF leads, as well as right bundle branch block (RBBB) (**Figure 1A**). The cardiac enzyme tests found that serum cTnI, creatine kinase isoenzyme MB (CK-MB), and myoglobin were positive. Based on these findings, the patient was initially diagnosed with acute inferior myocardial infarction. The patient underwent urgent coronary angiography (CAG) since the authorized family member signed the informed consent. CAG revealed subtotal occlusion of the middle segment in the right coronary artery (RCA) and 30%–80% diffuse stenosis of the proximal to middle section in the left anterior descending branch (LAD). PCI was performed immediately, and blood flow returned to TIMI III level after implantation of two rapamycin drug-coated stents in RCA (**Figures 1C, D**).

Postoperative echocardiography suggested localized thinning of the anterolateral and inferior walls of the left ventricle as well as the basal to mid-segment of the inferior interventricular septum, with reduced amplitude of motion. Additionally, cardiac ultrasound also detected left ventricular systolic dysfunction with a reduced ejection fraction of 46%, mild-to-moderate tricuspid regurgitation, and mild pulmonary artery hypertension (PAH) with 40 mmHg of pulmonary artery systolic blood pressure. Postoperatively, the patient was administered with aspirin at 100 mg once daily and ticagrelor at 90 mg twice daily for antiplatelet and rosuvastatin at 10 mg once daily for lipid-lowering therapy. To improve post-infarction heart failure, the patient was also given guideline-directed medical therapy (sacubitril/valsartan 50 mg twice daily, dagliflozin 10 mg once daily, metoprolol succinate 23.75 mg once daily, and spironolactone 20 mg once daily). A routine test of cardiac enzyme on the first and third postoperative days did not show a downward trend of cTnI. The repeat ECG did not show significant changes from the previous result. As before, the serum cTnI levels remained high for several days afterward (**Supplementary Table S1**). In addition, the patient’s serum CK-MB and myoglobin levels also did not decline. However, the patient did not complain any chest discomfort and refused to undergo further examination. On the 7th postoperative day, the patient was discharged without any special discomfort.

**Figure 2:**
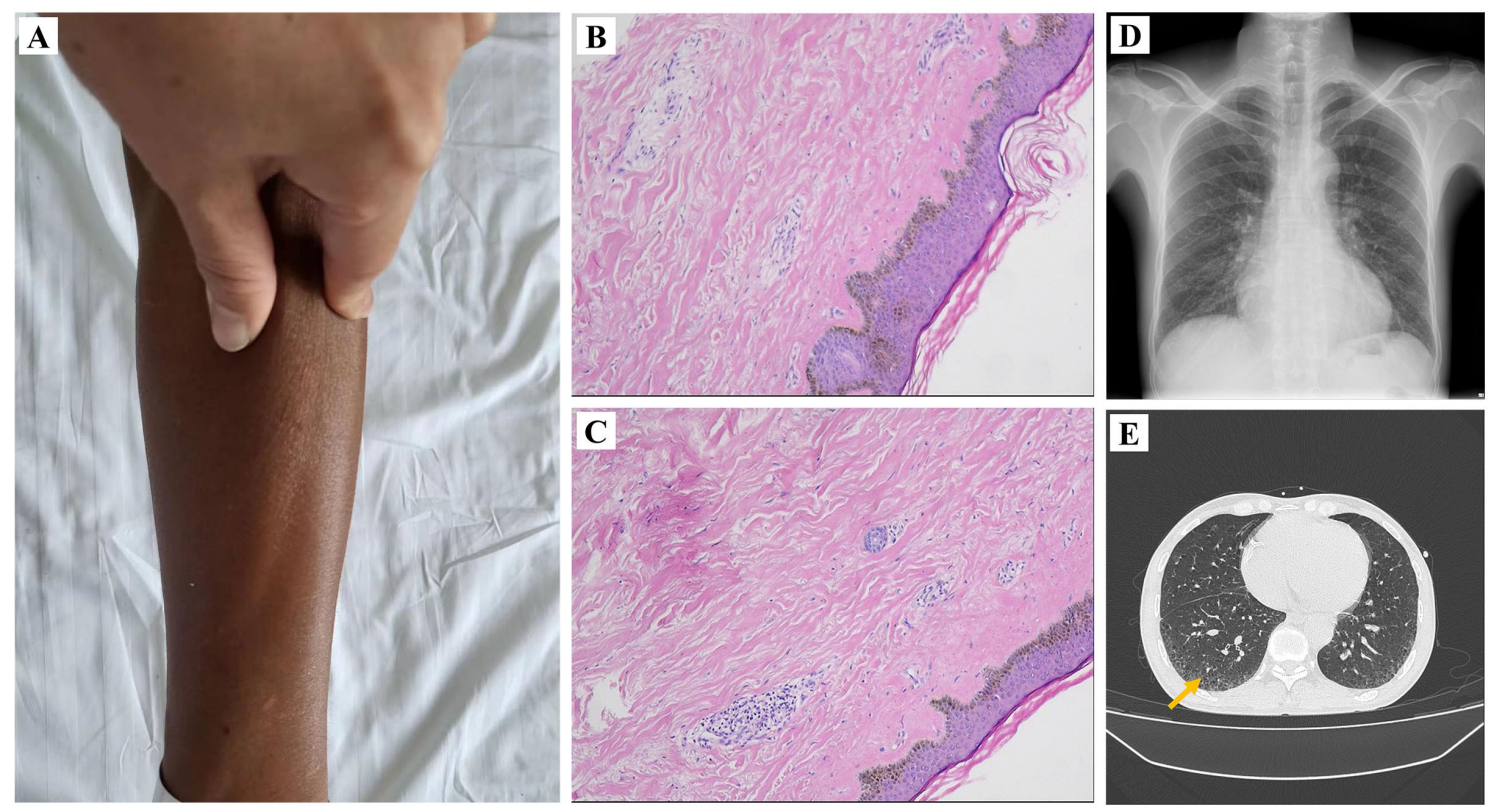
Features of skin appearance and pathology as well as lung imaging findings. The skin on the patient’s forearm presented as tight and dull **(A)**. The pathological findings indicated dermal and subcutaneous thickening with fibrous tissue hyperplasia and focal chronic inflammation **(B, C)**. Chest X-ray revealed thickened lung texture and slight enlargement of the right heart **(D)**. Chest computed tomography (CT) showed pulmonary fibrosis in the right lower lung (superior segment) **(E)**.

After 1 month, the patient was readmitted for treatment of residual vasculopathy. There was no significant change on ECG from the last hospitalization (**Figure 1B**). The patient underwent the second PCI with one stent implantation in the middle segment of the LAD (**Figures 1E, F**). The preoperative and postoperative tests of serum cTnI showed that it remained at a high level as well as serum CK-MB and myoglobin (**Supplementary Table S2**). After carefully inquiring about medical history again, the patient complained of no specific discomfort in the past except for skin tightness in the extremities, shortness of breath after exercise, and fingertip pain in cold weather. A detailed physical examination revealed that the patient had dark, stiff skin on both upper extremities, especially on the forearms (**Figure 2A**). However, the patient lacked any other special manifestations in the hands, such as finger ulcers and issues of skin edema, thickening, and hardening (**Supplementary Figure S1**). Additionally, the patient did not exhibit facial butterfly rash or joint swelling and pain. Hence, in response to the abnormal results of the physical examination and laboratory tests, a series of tests were arranged to clarify the presence of comorbid connective tissue and autoimmune system diseases.

**Figure 3:**
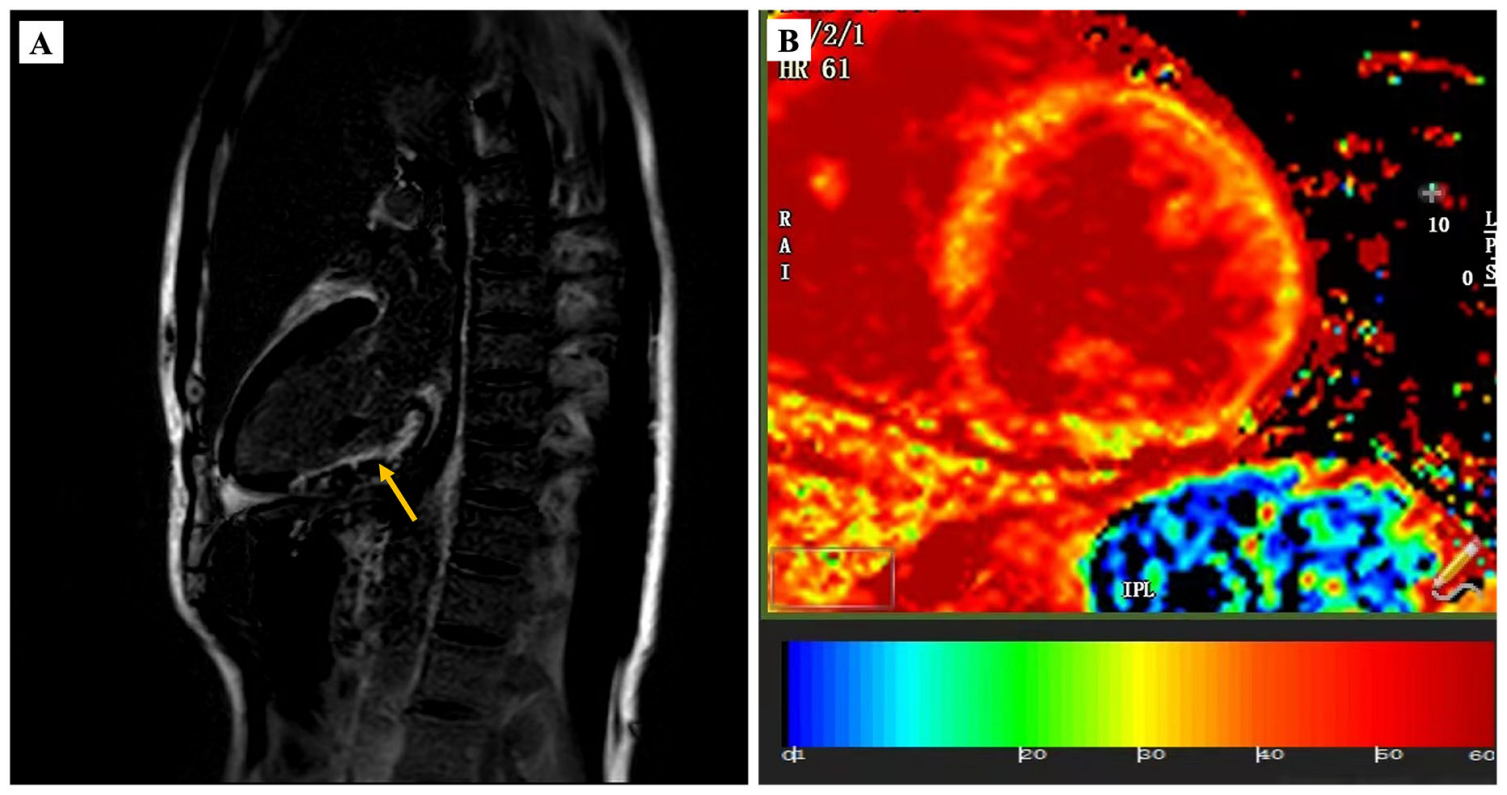
CMR characteristics of patient during the second hospitalization. LGE-verified myocardial fibrosis in inferior wall subendocardial (as shown by a yellow arrow) **(A)**. T1 mapping ECV confirmed severe myocardial fibrosis **(B)**.

Serum antinuclear antibody (ANA) was positive, but serum anticentromere antibody (ACA) and anti-topoisomerase I antibodies (anti-Scl-70 antibody) were negative which are the specific indicators of SSc. Slightly accelerated erythrocyte sedimentation rate was detected at the same time. Furthermore, another muscle injury indicator, creatine kinase, was also found to be significantly higher than normal. Moreover, to further aid in differential diagnosis, we also tested specific autoantibodies associated with other autoimmune diseases such as systemic lupus erythematosus (SLE) and rheumatoid arthritis (RA), all of which revealed negative results (**Supplementary Table S2**). Chest X-ray suggested thickened lung texture and slightly enlargement of the right heart (**Figure 2D**). Computerized tomography (CT) of chest suggested limited pulmonary fibrosis in the dorsal segment of the lower lobes of both lungs (**Figure 2E**). Cardiac magnetic resonance(CMR)test was performed to further define the myocardial damage. In addition to late gadolinium enhancement (LGE) in the inferior wall subendocardial myocardium, more diffuse myocardial fibrosis was explored by significantly higher native T1-mapping and extracellular volume (ECV) (**Figures 3A, B**). Due to the negative results for specific autoantibodies associated with SSc, skin biopsy was performed for a definitive diagnosis. The results of a histologic examination suggested dermal and subcutaneous tissue thickening with fibrous tissue hyperplasia, reduced cutaneous appendages, and focal chronic inflammation, which were consistent with the skin pathological manifestation of SSc (**Figures 2B, C**). The patient was ultimately diagnosed with systemic sclerosis, systemic sclerosis with cardiac and pulmonary involvement as well as prior MI.

**Figure 4:**
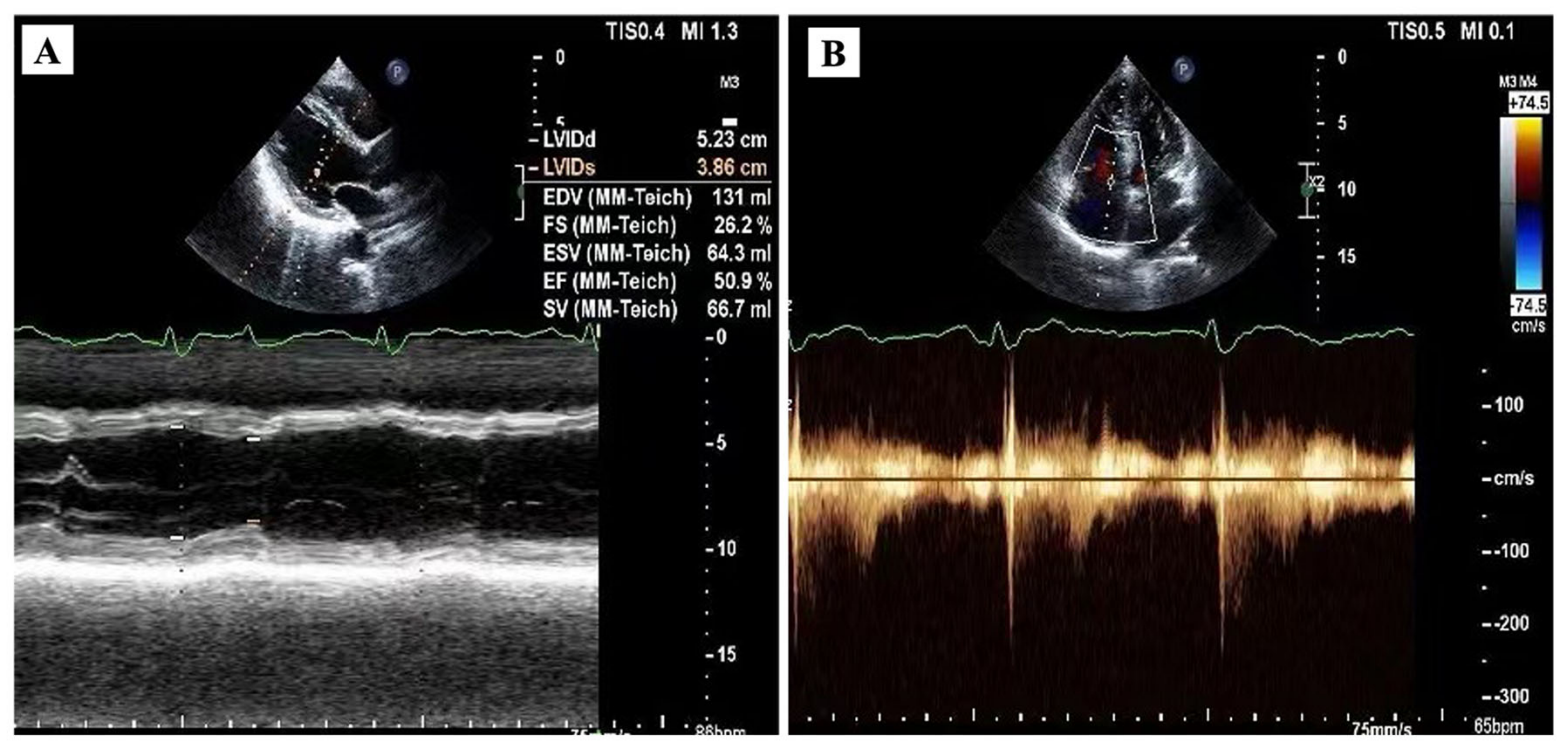
Echocardiogram during outpatient follow-up. Left ventricular ejection fraction was increased at 3 months after discharge **(A)**. The tricuspid regurgitation was alleviated as well **(B)**.

After consultation at the rheumatology department, the patient was treated with prednisone, cyclophosphamide, and mycophenolate mofetil based on dual antiplatelet, lipid lowering, and improved cardiac remodeling therapy. At 3 days after diagnosis confirmation, the patient was discharged, and the medication was instructed to be continued. At 3 months after discharge, the patient’s outpatient review showed that the cardiac function had improved significantly, and the serum cTnI level had dropped into the normal range (**Figures 4A, B**). Furthermore, the patient claimed that shortness of breath and skin tightness were alleviated after medication.

## Discussion

Acute myocardial infarction is a serious life-threatening cardiovascular disease, which is an event of myocardial necrosis caused by unstable ischemia syndrome (2). The recognized pathogenesis of AMI is the erosion and rupture of lipid-loaded coronal atherosclerotic vulnerable plaques and subsequent thrombosis formation on top of it (6). Cardiac troponin (cTn) is a pivotal biomarker for diagnosing myocardial infarction. Enzyme kinetics following AMI have been demonstrated to correlate with prognosis. CK kinetics demonstrate an increase in plasma within 3 to 5 h after AMI onset, reaching its peak at 16 to 20 h and returning to normal levels within 48 to 72 h. The release of cTn commences later than CK-MB. The plasma cTnI levels rise significantly, reaching an early peak at 6 h post-reperfusion and declining thereafter at 72 h (7). The clinical half-life of cTn following MI ranges from 7 to 20 h; however, recent findings indicate that the elimination T^1/2^ of cTnI and cTnT is 5 to 16 h shorter than previously reported (8). Previous studies have shown that asymptomatic rises of cardiac enzymes can occur during several hours after successful revascularization procedures (9). In this case, cardiac biomarker testing revealed persistently elevated cTnI levels when the patient was readmitted at 1 month after the initial PCI.

In the past decade, cTnI has been regarded as the gold-standard biomarker for detecting acute myocardial necrosis, which is the classical pathological feature of AMI (10). Typical rising of cardiac biomarker including cTnI might suggest early stent thrombosis, especially within 48 h after PCI (11). It is generally recognized that cTnI is exclusive to myocardial tissue, but it is also expressed highly in some extreme cases, such as in idiopathic inflammatory myopathies (IIM) and SSc (12). CK-MB is also a specific indicator for the diagnosis of myocardial injury and can be used to diagnose myocardial infarction, but it is necessary to rule out skeletal muscle injury. CK-MB levels have been reported to be significantly higher in SSc sufferers, especially those with a combination of higher serum ferritin (13). Additionally, elevated serum myoglobin can be detected in patients with a range of rheumatic diseases, including SSc (14).

SSc is a rare autoimmune disease of unknown etiology characterized by inflammation, vasculopathy, and ultimately leading to extensive fibrosis of skin as well as various internal organs (15). Due to lack of idiosyncratic manifestation, early diagnosis of SSc is not easy, especially in combination with other acute and critical conditions. Previously, there have been some reports of ACS or AMI combined with SSc. Edward T. Carreras and his colleague reported a case that a middle-aged woman presented as ACS resulting from coronary vasculitis with a distinct history of SSc and prior left circumflex PCI (16). Another article elucidated a SSc patient who developed anti-mitochondria antibody-positive primary biliary cirrhosis following AMI (17). The main difference between these cases and ours is that these patients had a clearly known history of SSc while suffering from ACS or AMI.

In our case, the abnormality of enzymatic indicators in the patient suggested that there might be a combination of some other muscle damage diseases, which cannot be explained by primary heart diseases. Previous studies confirmed that cardiac troponin T concentration was significantly increased in SSc patients (18, 19). Furthermore, other scholars had reported that high-sensitivity cTnI level was obviously higher in SSc patients than the control population (20). This indicates that cTnI may also be a potential cardiac biomarker in SSc. The patient tested positive for anti-nuclear antibodies (ANA), which can be frequently detected in 85%–99% of SSc patients (21). However, ANA could also be found in other rheumatic diseases, thereby lacking diagnostic specificity for SSc. We also tested the patient for ACA and anti-Scl-70, which are specific biomarkers for SSc, but the results were negative for both. The most common autoantibodies in SSc are ACA and anti-Scl-70; nevertheless, the positivity rates for both of these autoantibodies are less than 50% (22). Additionally, SSc is characterized by excessive skin fibrosis, particularly affecting the hands and the upper limbs. These classic changes encompass finger ulcers, skin edema, thickening, and hardening, presenting as stiffness, joint pain, tendinitis, and joint contractures (23). Generally, the cutaneous manifestations of SSc typically progress through three stages: (1) edematous phase, (2) indurative phase, and (3) atrophic phase. During the indurative phase, the skin on the distal parts of the limbs thickens and tightens, developing a taut, glossy, waxy appearance (24). The abovementioned skin manifestations may persist throughout the entire course of the disease. However, it should be noted that some patients may exhibit individual variations in disease presentation. In our case, the patient lacks these typical hand characteristics, presenting primarily with skin pigmentation and stiffness in the forearms of the upper limbs. Skin biopsy has been recommended for the diagnosis of SSc. Distinctive features of skin histology in SSc include thickening of the epidermis to subcutaneous tissue, increased thickness and hardening of dermal collagen bundles, loss of peripheral attachment tissue fat and hair follicles, as well as minimal plasma cell and lymphocyte infiltration (25). In the present case, the skin histologic manifestations of the patient were highly in line with SSc. Consequently, the patient was diagnosed with MI combined with SSc.

Cardiac involvement is the critical reason of mortality in SSc, which is responsible for almost 27.2% of all SSc-related deaths (26). The main cardiac abnormalities associated with SSc include conduction system abnormalities, malignant arrhythmia, left ventricular dysfunction, and focal or extensive myocardial fibrosis (5). Additionally, pulmonary artery hypertension (PAH) can be often detected in SSc patients and suggests poor survival in sufferers (27, 28). The main presentation of arrythmia in this patient was RBBB, which can be detected in approximately 27% of SSc patients and is an independent predictor of poor prognosis in SSc (29). CMR has been widely used in the evaluation of heart involvement in rheumatic diseases, which can reveal myocardial lesions especially in the absence of symptoms or positive results of cardiac ultrasound (30). Among this, T1 mapping and ECV quantification are the perfect indicators reflecting diffuse myocardial fibrosis, which could discover subclinical myocardial involvement in asymptomatic SSc individuals (31). In our case, CMR demonstrated that diffuse myocardial fibrosis existed in the patient by T1 mapping and high ECV, which were highly in accordance with the manifestations of SSc heart involvement.

In the past, macrovascular diseases are considered extremely rare in the heart, even though it is the major organ involved in SSc (32). However, in a large cohort study of patients with SSc, it was identified that there was a threefold increase in the prevalence of coronary artery diseases (CAD) compared to the general population (33). Additionally, some scholars found that atherosclerotic plaque lesions can occur in the absence of intima-media thickening in both SSc and SLE patients (34). Hyperlipidemia is one of the core mechanisms underlying atherosclerotic disease. Nevertheless, in our case, the patient’s lipid levels were not elevated. During the initial hospitalization, both total cholesterol and low-density lipoprotein cholesterol were within normal ranges, and the triglycerides were at a low level (**Supplementary Table S1**). Following the initial hospitalization, the patient took statin medication regularly for lipid-lowering therapy. Subsequent lipid testing after readmission revealed a significant reduction in lipid levels compared to the baseline. A growing body of evidence now supports a causal relationship between elevated lipoprotein(a) [(Lp(a)] levels and atherosclerotic cardiovascular disease (ASCVD) (35, 36). Hence, we also measured the patient’s serum Lp(a) level during his initial hospitalization, which was found to be below 30 mg/dL (37). In addition, to determine whether the patient had concomitant peripheral artery and carotid atherosclerosis, we performed ultrasound examinations of the patients’ extremity and neck vessels. The results of the extremity vascular ultrasound revealed no significant signs of peripheral atherosclerosis. However, the carotid ultrasound showed bilateral intima–media thickening of the carotid arteries, along with atherosclerotic plaques in the left carotid artery and right subclavian artery (**Supplementary Figure S2**).

To sum up, the patient’s final diagnosis was AMI (the most severe form of coronary atherosclerotic heart disease) complicated by SSc. Regarding the pathogenesis, whether these two diseases share common molecular mechanisms and specific interactions requires further in-depth research to elucidate.

## Conclusion

SSc is a rare autoimmune disease with poor prognosis, on top of which a combination of myocardial infarction may further deteriorate the patient’s condition. Our case provides a new perspective on the diagnosis and treatment of SSc in combination with AMI, especially in patients with persistently high cardiac enzyme levels after PCI.

## Data Availability

The datasets presented in this study can be found in online repositories. The names of the repository/repositories and accession number(s) can be found in the article/**Supplementary Material**.
