# Supplementary material for: Case Report: Unusual persistent elevation of troponin I-systemic sclerosis masked by acute myocardial infarction
Source: Front Immunol. 2026 Feb 5;17:1675907. doi: 10.3389/fimmu.2026.1675907 (PMC12917895; doi:10.3389/fimmu.2026.1675907)
Supplement: Supplementary file 1 [file Image1.pdf]

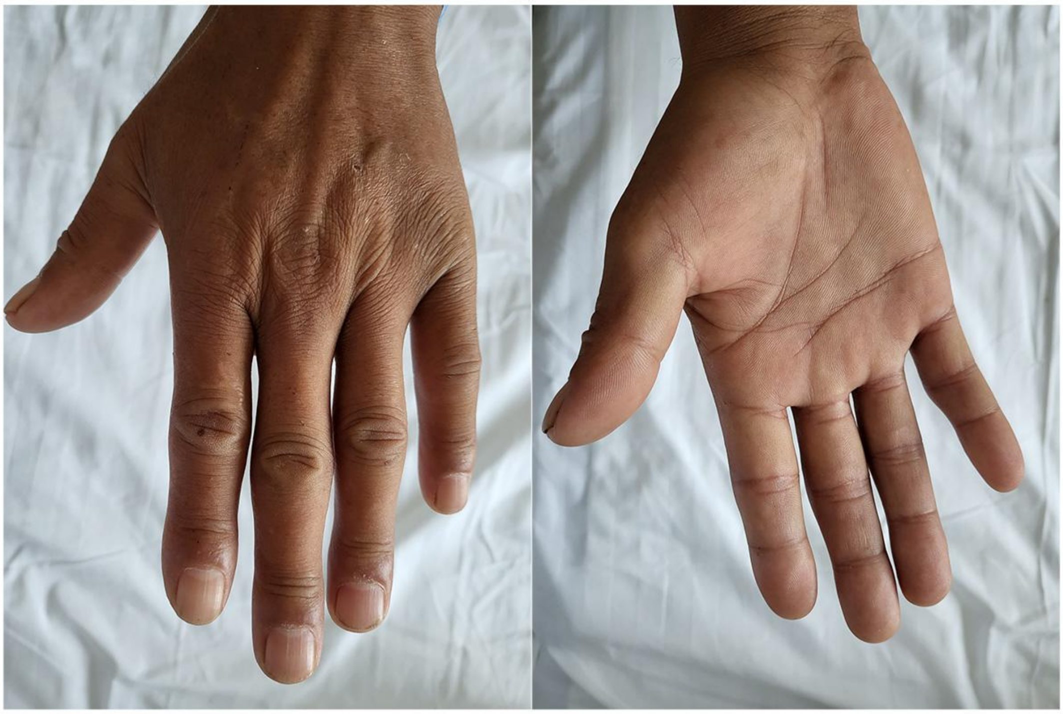

Figure S1. The hand’s skin manifestations of the patient.

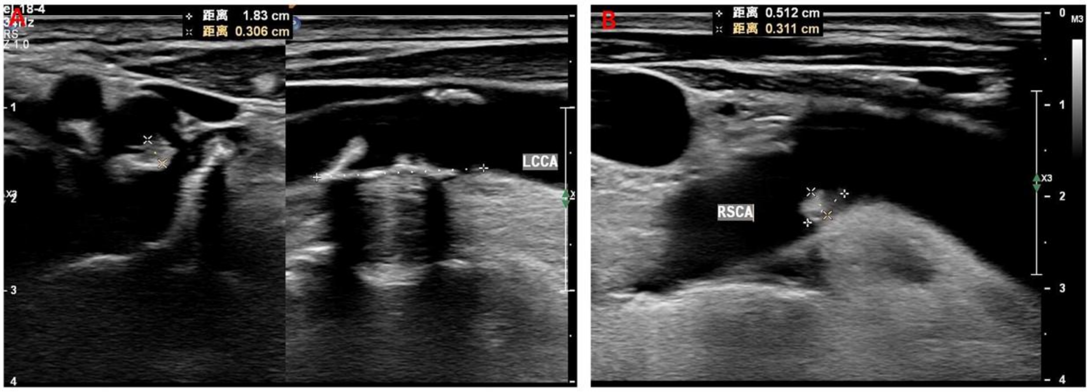

Figure S2. The patient’s carotid ultrasound results. **(A)** A highly echogenic plaque measuring approximately 18.3 × 3.1 mm was detected at the bifurcation of the left carotid artery. **(B）** Carotid ultrasound revealed a highly echogenic plaque (approximately 5.1 × 3.1 mm in size) on the posterior wall of the initial segment of the right subclavian artery.
