# Supplementary material for: Case Report: Unusual persistent elevation of troponin I-systemic sclerosis masked by acute myocardial infarction
Source: Front Immunol. 2026 Feb 5;17:1675907. doi: 10.3389/fimmu.2026.1675907 (PMC12917895; doi:10.3389/fimmu.2026.1675907)
Supplement: Supplementary file 2 [file DataSheet1.pdf]

Table 1. Laboratory data of the patient in the first admission

Variable	Reference Range (Adult)	Pre-operation	Post-operation Day 1	Day 3	Day 5	Day 7
Hemoglobin (g/L)	130-175	135	131	133		
Hematocrit (%)	40.0-50.0	41.6	41.1	41.2		
Platelet count (10^9/L)	125-350	221	228	223		
White-cell count (10^9/L)	3.50-9.50	8.19	7.55	7.44		
Neutrophils (%)	40.0-75.0	57.0	59.9	68.7		
Monocytes (%)	3.0-10.0	9.8	8.7	7.7		
C-reactive protein (mg/L)	0-4.00	4.90	5.31	5.01		4.16
Total protein (g/L)	65-85	67.0				
Albumin (g/L)	40-55	38.9				
Total Bilirubin (μmol/L)	0-23	6.7				
Direct Bilirubin (μmol/L)	0-4	1.5				
Aspartate aminotransferase (U/L)	15-40	70				
Alanine aminotransferase(U/L)	9-50	51				
Urea nitrogen (mmol/L)	3.1-8.0	5.93	6.21			
Creatinine (μmol/L)	57-97	57.6	69.3			

Total cholesterol (mmol/L)	3.6-5.7	2.84				2.39
Low density lipoprotein (mmol/L)	1.55-3.7	1.82				1.71
Triglyceride (mmol/L)	0.8-1.8	1.16				1.15
Lp(a) (mg/dL)	0.0-40.0	21.6				
Troponin I (ng/ml)	0.010-0.023	0.35	0.37	0.36	0.38	0.42
Creatine kinase MB isoenzymes (ng/ml)	2.0-7.2	67	51	49	60	68
Myoglobin (ng/ml)	23-112	329	400	513	558	582
NT-pro-BNP (pg/ml)	300-900	1420	1800	1298	934	785
