# Supplementary material for: Case Report: Unusual persistent elevation of troponin I-systemic sclerosis masked by acute myocardial infarction
Source: Front Immunol. 2026 Feb 5;17:1675907. doi: 10.3389/fimmu.2026.1675907 (PMC12917895; doi:10.3389/fimmu.2026.1675907)
Supplement: Supplementary file 3 [file DataSheet2.pdf]

Table 2. Laboratory data of the patient in the readmission

Variable	Reference Range (Adult)	Pre-operation	Post-operation	
			Day 1	Day 3
Hemoglobin (g/L)	130-175	131	128	
Hematocrit (%)	40.0-50.0	40.3	39.4	
Platelet count (10^9/L)	125-350	227	221	
White-cell count (10^9/L)	3.50-9.50	5.63	6.21	
Neutrophils (%)	40.0-75.0	65.8	67.9	
Monocytes (%)	3.0-10.0	6.9	6.5	
C-reactive protein (mg/L)	0-4.00	2.63	3.5	
Total protein (g/L)	65-85	65.5		
Albumin (g/L)	40-55	40.1		
Total Bilirubin (μmol/L)	0-23	4.9		
Direct Bilirubin (μmol/L)	0-4	1.4		
Aspartate aminotransferase (U/L)	15-40	63		
Alanine aminotransferase (U/L)	9-50	46		
Urea nitrogen (mmol/L)	3.1-8.0	6.33	3.59	
Creatinine (μmol/L)	57-97	62.5	62.8	
Total cholesterol (mmol/L)	3.6-5.7	2.25		
Low density lipoprotein (mmol/L)	1.55-3.7	1.35		
Triglyceride (mmol/L)	0.8-1.8	1.61		

Antinuclear antibody (ANA)	negative	positive
Scl-70 antibody	negative	negative
anticentromere antibody (ACA)	negative	negative
anti-nRNP/Sm antibodies (1:100)	negative	negative
anti-Sm antibodies (1:100)	negative	negative
anti-SS-A antibodies (1:100)	negative	negative
anti-Ro-52 antibodies (1:100)	negative	negative
anti-SS-B antibodies (1:100)	negative	negative
anti-Jo-1 antibodies (1:100)	negative	negative
PM-SCL antibodies (1:100)	negative	negative
anti-centromere protein B antibody (1:100)	negative	negative
proliferating cell nuclear antigen antibody (1:100)	negative	negative
anti-Nuclesome antibody (1:100)	negative	negative
anti-histone antibodies (1:100)	negative	negative
anti-ribosomal P protein antibodies (1:100)	negative	negative
anti-mitochondrial antibodies M2 (1:100)	negative	negative
Immunoglobulin IgM(g/L)	0.3-2.20	2.63
Immunoglobulin IgG(g/L)	8.6-17.4	16.6
Immunoglobulin IgA(g/L)	1.00-4.20	1.53
Complement C3 (g/L)	0.700-1.400	0.783

Complement C4 (g/L)	0.100-0.400			0.215
Rheumatoid Factor (IU/mL)	0-20.0			<20
Anti-cyclic Citrullinated Peptide Antibody (RU/mL)	0-12.0			4.6
erythrocyte sedimentation rate(mm/h)	0-15			23
Creatine kinase (U/L)	50-310			1482
Troponin I (ng/ml)	0.010-0.023	0.26	0.31	0.3
Creatine kinase MB isoenzymes (ng/ml)	2.0-7.2	66	50	48
Myoglobin (ng/ml)	23-112	405	424	439
NT-pro-BNP (pg/ml)	300-900	864	882	811
